# Implications of Cognitive Impairment on Antihypertensive Medication Use in HIV

**DOI:** 10.3390/v17040470

**Published:** 2025-03-26

**Authors:** Azin Tavasoli, Bin Tang, Mohammadsobhan S. Andalibi, Donald R. Franklin, Scott L. Letendre, Robert K. Heaton, Ronald J. Ellis

**Affiliations:** 1 Department of Neurosciences, University of California San Diego, San Diego, CA 92093, USA; atavasoli@health.ucsd.edu (A.T.); mandalibi@health.ucsd.edu (M.S.A.); 2 Department of Psychiatry, University of California San Diego, San Diego, CA 92093, USA; bit001@ucsd.edu (B.T.); dofranklin@health.ucsd.edu (D.R.F.); rheaton@health.ucsd.edu (R.K.H.); 3 Department of Medicine, University of California San Diego, San Diego, CA 92093, USA; sletendre@ucsd.edu

**Keywords:** HIV, cognition, antihypertensive medications, anticholinergic burden score

## Abstract

Background: Aging-related comorbidities such as cardiovascular disease and neurocognitive impairment are more common among people with HIV (PWH). Hypertension (HTN) has been implicated in cognitive decline, and antihypertensives with anticholinergic properties may exacerbate this decline. Our research probed the relationship between neurocognitive performance and antihypertensives in hypertensive PWH and in those without HIV (PWoH), examining whether increased antihypertensives followed the worsening in neurocognitive performance. Methods: This longitudinal analysis encompassed seven visits over five years, enrolled between 1999 and 2022. Participants were included if they reported HTN or used antihypertensives. All participants underwent comprehensive cognitive assessments, and their global cognitive performance was evaluated using summary, demographically corrected T-scores. The association between the global T-score and the number of antihypertensives was evaluated using generalized linear mixed-effects models. Summary regression-based change score (sRCS) was analyzed as an indicator of global performance over time. Results: Among 1158 hypertensive PWH (79.9% were on ART), worsening cognitive performance was associated with an increased number of antihypertensives (*p* = 0.012) but not in PWoH (*p* = 0.58). PWH had lower mean arterial pressure (MAP) than PWoH after adjusting for demographics (β = −5.05, *p* = 2.3 × 10^−11^). In PWH, an association between mean arterial pressure (MAP) and sRCS suggested that those with cognitive improvement had lower MAP (*p* = 0.027). PWH taking more anticholinergics were more likely to have worse cognitive performance over time (*p* < 0.001). Conclusions: PWH with declining neurocognitive performance over time used increasing numbers of antihypertensives, suggesting that their providers prescribed more antihypertensives because of either treatment refractory HTN or poor adherence. Prescribers should avoid using antihypertensives with anticholinergic properties when possible.

## 1. Introduction

Antiretroviral therapy (ART) has significantly increased the lifespan of people with HIV (PWH) [1]. By the year 2030, 70% of PWH will have reached the age of 50 or older [2]. Despite the success of ART in achieving durable virologic suppression, PWH are at increased risk for multiple comorbidities associated with aging in the general population, including cardiovascular disease (CVD) [3,4], lung disease, liver disease, kidney disease, diabetes, neurocognitive disorders, and other diseases [5,6]. Hypertension (HTN), characterized by systolic blood pressure (SBP) of 140 mmHg or higher and diastolic blood pressure (DBP) of 90 mmHg or higher [7], impacts one billion people globally, and its occurrence notably rises with advancing age [8]. Two-thirds of individuals aged over 60 are affected by hypertension, which markedly elevates the likelihood of developing vascular cognitive impairment [9].

The factors contributing to hypertension in PWH result from the interplay between risk factors seen in the general population and unique characteristics associated with the HIV environment [10]. Potential reasons for the elevated prevalence of hypertension in PWH encompass chronic inflammation, renal disease, blood vessel damage due to prolonged exposure to ART, and increased levels of behavioral risk factors in PWH [11]. In untreated HIV, these factors encompass immune deficiency, immune activation, and chronic inflammation, which may endure even after the initiation of ART. Furthermore, in treated HIV, elements of ART can directly influence blood pressure levels or indirectly affect them through ART-induced alterations in body composition [12].

Metabolic syndrome (MetS) frequently occurs in PWH [13]. MetS encompasses metabolic risk factors: abdominal obesity, atherogenic dyslipidemia, elevated blood pressure, and insulin resistance [14]. There has been a consistent association between MetS and neurocognitive impairment (NCI) as well as cognitive decline in the broader population [15,16]. When considering individual components of MetS in PWoH, hyperglycemia and hypertension typically display the most robust associations with NCI [17,18]. Nonetheless, as the number of prescribed medications in the context of multi-morbidity exceeds those prescribed for individual conditions, adherence may be comparatively suboptimal within this population [19]. Hypertension is associated with diminished performance on cognitive tests [20]. Previous studies found that low treatment adherence is also linked to suboptimal cognitive performance [21,22]. Vinyoles et al. [23] examined how cognitive impairment in individuals with hypertension relates to medication adherence. Their findings revealed that patients with cognitive impairment were more prone to having inadequately controlled blood pressure, displaying higher rates of non-adherence. Additionally, they were more inclined to receive combined drug therapy as opposed to monotherapy [23]. Individuals diagnosed with hypertension elevate their susceptibility to Alzheimer’s disease (AD) and vascular dementia, with as high as 60% of hypertensive patients experiencing compromised cognitive function [24]. Furthermore, hypertensive individuals with lower cognitive scores on the Mini-Mental State Examination exhibited a sixfold higher likelihood of non-compliance with antihypertensive medication in comparison to individuals with normal scores [25].

Numerous elderly individuals regularly use anticholinergic medications over an extended period, and prolonged usage has been linked to cognitive decline and dementia [26]. These medications have been correlated with an increased presence of amyloid plaques in the brains of individuals diagnosed with Parkinson’s disease [27]. The anticholinergic burden of prescribed medications strongly predicts cognitive and physical impairments in older individuals [28]. The anticholinergic cognitive burden scale, developed by Boustani et al., derives from a comprehensive literature review on medications exhibiting anticholinergic properties. This scale encompasses medications deemed to affect cognition potentially detrimentally [29,30]. Some medications used to treat hypertension exhibit anticholinergic properties [27].

In the current longitudinal cohort study, we investigated the hypothesis that neurocognitive performance is associated with higher numbers of antihypertensive medications. Furthermore, we explored whether this association varied between PWH and PWoH. Our study aimed to assess the impact of neurocognitive performance on the number of prescribed antihypertensive medications while considering the participants’ HIV status. We also evaluated the effect of cognitive impairment on mean arterial pressure (MAP).

## 2. Results

### 2.1. Participant Characteristics

At baseline, the 1158 PWH diagnosed with HTN were predominantly middle-aged (mean 49.7 years), with the majority being white (49.1%) and male (83.3%). Also at baseline, 938 (79.9%) were on antiretroviral therapy (ART); 738 (67.3%) had plasma HIV RNA ≤ 200 copies/mL; and 70.2% of participants had a documented history of AIDS-defining conditions as per CDC classification criteria. (Table A1) PWoH with HTN (*n* = 272) were older (mean 55.2 years) at baseline and were more likely to be female (34.6%) and white (58.8%). 59.2% were prescribed antihypertensive medications [Table 1].

At the baseline visit, there was no significant difference in cognitive status between ARV-naïve individuals and PWH receiving either HAART or non-HAART treatment. At the baseline visit, a history of CNS infections in PWH, including progressive multifocal leukoencephalopathy (1.60%), toxoplasmosis of the brain (2.53%), and neurosyphilis (0.03%) was noted.

### 2.2. Association of Cognition and Antihypertensive Medication over Time

The results in Table 2 showed that every 10-unit increase in global T-score (better cognitive function) was associated with a decrease of 8.4% in antihypertensive count; that is, a higher global T-score was associated with lower antihypertensive medication count in PWH (*p* = 0.027). The significant association remained after adjusting for covariates in multivariable analysis (*p* = 0.012). Global T-score was not associated with the antihypertensive count in PWoH (*p* = 0.50).

Out of 135 participants who increased their antihypertensive medication between the baseline visit and the follow-up visits over five years, 107 (80%) were PWH.

### 2.3. Association of Global Cognitive Change Score (sRCS) and MAP

**Figure 1:**
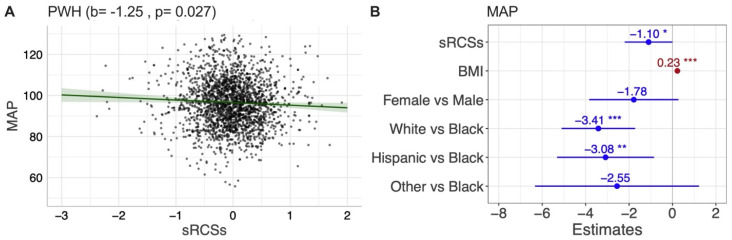
Association between MAP and sRCS in univariable (**A**) and multivariable analyses in PWH (**B**) sRCS: summary regression change score; MAP: mean arterial pressure. * *p* < 0.05, ** *p* < 0.01, *** *p* < 0.001.

We also found that PWH had lower MAP than PWoH over time after adjusting for demographics (β = −5.05, *p* = 2.3 × 10^−11^). The results showed that higher sRCS was associated with lower MAP; that is, those with better cognitive change (greater cognitive improvement or less cognitive decline) were more likely to have lower MAP in PWH (see Figure 1A, *p* = 0.027). The sRCS remained significantly associated with MAP after controlling for covariates (*p* = 0.0498). In addition, higher BMI was associated with higher MAP, while White and Hispanic people were more likely to have lower MAP compared to Black people (see M4 in Table 3 and Figure 1B).

### 2.4. Association of Cognition and Total Anticholinergic Medications (Including Antihypertensives) over Time

In a multivariable analysis, an increasing number of anticholinergic medications in PWH was associated with a lower global T-score over time (*p* < 0.001), while no evidence supported the association in PWoH (*p* = 0.68) (Table 4). Blacks had better cognitive performance (higher global T-score) than other races in people with and without HIV.

Additionally, we observed a highly positive correlation between the number of anticholinergics and antihypertensive medications in PWH (b = 0.45, *p* = 0.001).

## 3. Discussion

This longitudinal study provides novel evidence that declining neurocognitive performance predicts increased antihypertensive medication requirements in PWH but not PWoH. Three key findings emerge: (1) PWH with declining NC performance required more antihypertensive medications over time; (2) cognitive improvement was associated with better blood pressure control (lower MAP), specifically in PWH; and (3) anticholinergic burden from antihypertensive medications was associated with worse cognitive outcomes in PWH. These findings suggest a complex bidirectional relationship between cognitive function and blood pressure control that may be uniquely modified by HIV status. Several potential mechanisms may explain these findings. First, HIV-associated neuroinflammation could simultaneously affect cognitive function and vascular regulation, creating a unique pathophysiological environment different from PWoH [31,32]. Second, chronic inflammation in PWH might accelerate vascular aging, affecting both cognitive function and blood pressure control [33]. Third, cognitive deficits might affect medication adherence and self-care, making it more challenging for individuals to manage hypertension effectively. The adherence of patients to prescribed medication, also known as compliance or concordance, stands out as a paramount therapeutic element [34]. In elderly patients, a connection between inadequate medication adherence and memory or other cognitive impairments has been observed. Forgetfulness has been identified as a causative factor in 16–40% of cases among the elderly [35,36]. A systematic review in the National Library of Medicine delves into the correlation between non-adherence to medication and distinct cognitive domains among individuals with cognitive impairment (CI). Another study, sourced from the National Library of Medicine, explores the connection between mild cognitive impairment (MCI) and non-adherence to medication in the elderly. This cross-sectional study probes into the influence of MCI on medication adherence and identifies MCI as a noteworthy factor contributing to non-adherence, with potential repercussions on disease outcomes [37]. Poor neurocognition might lead to irregular or missed doses, resulting in less effective blood pressure control [38]. Poor neurocognition has been linked to adverse everyday functioning and health-related outcomes—e.g., unemployment, medication adherence, and healthcare management, which over time might necessitate more antihypertensive medications to achieve adequate control. The differential impact of changes in antihypertensive medications on changes in cognitive performance between PWH and PWoH warrants further exploration. For example, we could speculate that the relationship is modified by persistent inflammation in PWH compared to PWoH, where inflammation is less. This could be evaluated by studying biomarkers and how they change over time in these two groups. Anti-retroviral medications might interact with anti-hypertensive medications, increasing or decreasing their concentrations and resulting in differential effects. This could imply underlying differences in how these medications affect cognitive outcomes across these populations, possibly influenced by HIV’s pathophysiology or interactions with antiretroviral therapy. Prior studies suggest low-copy viremia has minimal impact on cognitive outcomes when viral suppression is generally maintained [39].

Anticholinergic drugs may also correlate with enduring cognitive impairment [40,41,42]. Many antihypertensive medications, such as captopril and furosemide, exhibit anticholinergic effects [43]. The observation that the anticholinergic effects of antihypertensive medications worsen cognitive decline underscores the need for a nuanced approach to prescribing and managing these drugs. Some antihypertensive agents have minimal or no anticholinergic effects, and these should be prioritized for use, especially in older individuals. Thus, prescribers should consider individual patient characteristics, including age, cognitive status, and medication history, when selecting antihypertensive agents. Furthermore, regular monitoring for cognitive changes and adjustment of medication regimens as needed is crucial to mitigate the risk of cognitive impairment while ensuring adequate blood pressure control.

Neurocognitive impairment might also influence an individual’s ability to make healthy lifestyle choices, such as maintaining a balanced diet, engaging in regular physical activity, and managing stress [44]. Insel and colleagues (2008) discovered a significant association between memory and executive function and medication adherence in a sample of 16 elderly individuals with hypertension [45]. A systematic review conducted by Smith and colleagues regarding medication nonadherence in individuals with dementia or cognitive impairment revealed suboptimal adherence rates, ranging from 10.7% to 38% for those with cognitive impairment. In contrast, adherence levels ranged from 17% to 100% among elderly individuals diagnosed with Alzheimer’s disease [46].

These findings have immediate implications for clinical practice. First, they suggest that cognitive screening should be integrated into routine care for PWH with hypertension, particularly when medication regimens become more complex. Second, clinicians should carefully consider the anticholinergic burden when selecting antihypertensive medications for PWH, potentially prioritizing agents with minimal anticholinergic effects. Third, interventions targeting cognitive function might improve blood pressure control, suggesting a novel therapeutic approach. Ethnicity might uniquely affect neurocognitive outcomes in PWV [47,48].

Several limitations should be considered when interpreting these findings. First, the reliance on self-reported hypertension and medication use may introduce recall bias, particularly relevant given the cognitive focus of this study. Second, while we adjusted for several confounders, unmeasured variables such as depression, stress, and socioeconomic factors might influence both cognitive function and medication use. Third, the study’s longitudinal nature may introduce survivor bias, potentially underestimating the true associations. Fourth, the generalizability of findings across different HIV subtypes, treatment regimens, and healthcare settings requires further investigation. Fifth, while comprehensive, the cognitive assessment tools may not capture all relevant aspects of cognitive function affecting medication management.

While adherence issues were raised as a possible explanation for the observed associations, a deeper discussion of vascular or pharmacokinetic mechanisms (e.g., differential effects of antihypertensives in HIV) would strengthen the argument. While major confounding medical conditions were excluded, acknowledging the potential influence of unexcluded CNS infections or tumors would improve transparency.

Future research endeavors should pursue a comprehensive approach encompassing mechanistic, clinical, and implementation studies to optimize hypertension treatment in PWH. Key priorities include investigating the biological mechanisms underlying cognition–blood pressure relationships through examination of inflammatory markers, vascular aging processes, and blood–brain barrier integrity while simultaneously conducting randomized clinical trials to evaluate cognitive interventions for blood pressure control and assess the impact of simplified medication regimens. Implementation research should focus on developing and validating integrated care models that combine cognitive and cardiovascular monitoring, supported by cost-effectiveness analyses and technology-based adherence support systems for cognitively impaired PWH. This coordinated research strategy will enhance our understanding of the complex interplay between HIV, cognition, and hypertension management, ultimately leading to more personalized and effective treatment approaches that improve patient outcomes.

### Public Health Implications

These findings have broader implications for healthcare policy and resource allocation. As the HIV population ages, the intersection of cognitive and cardiovascular health will become increasingly important. Healthcare systems may need to adapt to provide more integrated care, potentially including routine cognitive monitoring and specialized support for medication management. The potential cost savings from prevented cardiovascular complications and reduced medication complexity could offset the resources required for cognitive screening and intervention. These considerations should inform future HIV care guidelines and healthcare planning.

## 4. Materials and Methods

### 4.1. Participants

CNS HIV Antiretroviral Therapy Effects Research (CHARTER) is an ongoing, prospective, observational study conducted at six US academic medical centers. The institutional review board at each site approved the research, and every participant provided written informed consent. We evaluated the longitudinal association of antihypertensive medications with neurocognitive (NC) performance for up to seven visits over five years in the CHARTER and HIV Neurobehavioral Research Center (HNRC) cohort enrolled between 1999 and 2022. At the follow-up visits, the neuromedical and neurobehavioral assessments encompassed all the elements of the initial baseline evaluation. Participants were included if they reported HTN or used antihypertensive medications at their baseline visit. Participants with severe medical (e.g., traumatic brain injury with more than 30 min of loss of consciousness) or psychiatric (e.g., psychosis) conditions that confounded attribution of the cause of NCI to HIV and/or prescribed medications were excluded prior to cognitive assessment [49]. Participants provided written informed consent for using their data in research.

### 4.2. Neurobehavioral Assessments

All participants underwent a thorough neurocognitive assessment, which included a comprehensive battery of tests targeting seven neurocognitive domains commonly impacted by HIV [50]. Raw test scores were transformed into T-scores adjusted for age, education, gender, race, ethnicity, and practice effects [51]. All domains’ T-scores were averaged to generate a global T-score.

To assess changes in neurocognitive performance from baseline to the follow-up visits, we calculated global cognitive change scores (z-scores) based on the regression equation generated from the norming group [51]. These z-scores represent a participant’s performance at follow-up, compared to what would be expected for someone with similar baseline NC and other relevant factors (e.g., age, education). The z-scores across all tests were averaged to generate a summary regression change score (sRCS), which can be considered as a continuous variable or as a method to characterize what would be an unusual test–retest change for clinically stable individuals. For the latter, the top 5% of the sRCS distribution in the normative sample is used to classify significant “improvement”, while the bottom 5% indicates a significant “decline”. The middle 90% were classified as “stable”.

### 4.3. Laboratory Assessment

HIV infection was identified by a commercial diagnostic test. At each medical center laboratory, certified under the Clinical Laboratory Improvement Amendments (CLIA) or equivalent certification, routine clinical chemistry panels, complete blood counts, rapid plasma regain tests, and CD4+ T cell counts (utilizing flow cytometry) were conducted. Plasma HIV RNA was quantified by reverse transcriptase–polymerase chain reaction (Roche Amplicor, version 1.5) with a lower limit of quantitation of 200 copies/mL. AIDS diagnosis was established based on accessible clinical and immunological data, specifically defined as having a nadir CD4 cell count of less than 200 cells/μL or any history of a clinical condition meeting the criteria for AIDS as per the CDC AIDS classification system [52].

### 4.4. Statistical Analysis

Statistical analyses were performed using JMP Pro (version 17, SAS, Cary, NC, USA) and R (version 4.4.0, 2024). If variable distributions were skewed, values were transformed as appropriate (e.g., log10 or square root transformation) to improve distribution normality before analyses. Demographic and clinical characteristics were summarized and presented as counts (percentages), averages (standard deviations), or medians (interquartile ranges). Categorical data were compared through the Chi-Square test. Group comparisons were performed using the independent sample *t*-test (for normally distributed data) or the Mann–Whitney test (for non-normally distributed data). Association between global T-score and antihypertensive medication count was assessed in PWH and PWoH separately using Poisson generalized linear mixed effect models (Poisson GLMM), adjusting for potentially confounding variables such as age, sex, ethnicity, body mass index (BMI), and potentially important HIV disease markers (i.e., nadir CD4 count and current HIV RNA levels). The effect of anticholinergic medications (antidepressants, antihistamines, antihypertensives, and other related medications) on global T-score was detected in mixed-effects models with both a random intercept and slope stratified by HIV status.

Association between sRCS and MAP was assessed in PWH and PWoH separately using linear mixed-effects models, adjusting for potentially confounding variables such as age, sex, ethnicity, BMI, and potentially important HIV disease markers. Table 5 shows outcomes, the main predictors, and potential covariates.

## 5. Conclusions

To summarize, this longitudinal study aimed to investigate the role of neurocognitive performance in managing blood pressure among PWH undergoing ART, compared to PWoH. The study found that PWH with declining neurocognitive function required more antihypertensive medications. These findings suggest that regular monitoring of neurocognitive function in PWH on antihypertensive therapy could help optimize medication use, potentially reducing the pill burden and minimizing drug–drug interactions. Enhancing cognitive function in PWH may decrease the need for multiple blood pressure medications, improving overall patient outcomes. PWH with cognitive impairment had higher MAP, suggesting blood pressure control may be linked to enhanced cognitive function.

## Figures and Tables

**Table 1:** Baseline demographics and clinical characteristics by HIV serostatus.

Variables	All (*n* = 1457)	PWH (*n* = 1158)	PWoH (*n* = 272)	*p*-Value *
**Demographic Characteristics**				
Age (years), M (SD)	50.7 (10.8)	49.67 (10.4)	55.15 (12.3)	**<0.0001**
Education (years), M (SD)	13.3 (2.84)	13.21 (2.79)	13.58 (3.01)	0.052
Male, N (%)	1171 (79.88%)	987 (83.29%)	178 (65.44%)	**<0.0001**
Race/Ethnicity, N (%)	-	-	-	**<0.0001**
-White, non-Hispanic	745 (50.85%)	581 (49.07%)	160 (58.82%)	
-Black	432 (29.42%)	383 (32.35%)	48 (17.65%)	
-Hispanic	242 (16.52%)	179 (15.12%)	(21.69%)	
-Other	46 (3.14%)	41 (3.46%)	5 (1.84%)	
On antihypertensive medication	711 (59.15%)	548 (59.51%)	156 (57.35%)	
-Beta blockers	234 (19.46%)	191 (20.73%)	42 (15.44%)	
-CCB	137 (11.34%)	95 (10.31%)	41 (15.07%)	0.055
-ARB	57 (4.75%)	38 (4.12%)	19 (7.00%)	
-ACE inhibitor	247 (20.47%)	183 (19.87%)	61 (22.42)	
-Alpha blockers	47 (3.68%)	41 (4.01%)	6 (2.00%)	
-Alpha agonist	32 (2.88%)	28 (3.27%)	4 (1.27%)	
-Diuretics	90 (7.48%)	74 (8.00%)	15 (5.55%)	
-Potassium sparing	30 (2.12%)	25 (2.11%)	5 (2.23%)	
-Combination therapy	204 (17.00%)	149 (16.17%)	53 (19.50%)	-
On anticholinergic meds	1114 (76.00%)	930 (78.51%)	177 (65.04%)	**<0.0001**
Neurocognitive impairment	612 (41.67%)	542 (44.21%)	84 (30.88%)	**<0.0001**
Global T-scores	46 (7.46)	45.7 (7.61)	47.8 (6.53)	0.004
Myocardial infarction	80 (5.49%)	71 (6.02%)	9 (3.33%)	0.082
Hyperlipidemia	517 (35.48%)	411 (34.68%)	106 (39.00%)	0.18
BMI, M (SD)	28 (6.41)	27.65 (6.11)	29.50 (7.36)	**0.0001**
**HIV Characteristics**				
Duration of HIV (years), M (SD)	-	14.4 (8.44)	-	
AIDS diagnosis, N (%)	-	828 (70.17%)	-	
Current CD4, median (IQR)	500 (276, 752)	462 (256, 699)	-	
Nadir CD4, median (IQR)	169 (36, 350)	127 (28, 270)	-	
ART ON, n (%)	-	938 (79.9%)	-	
Plasma HIV RNA ≤ 200 copies/mL, n (%)	-	738 (67.3%)	-	

* The chi-square and independent samples *t*-test were used to compare categorical variables and group means, respectively. CCB: calcium channel blockers; ARB: Angiotensin II receptor blockers; ACE: angiotensin-converting enzyme.

**Table 2:** Associations between antihypertensive medication count and global T-score and demographic and clinical variables using Poisson GLMM, stratified by HIV status.

	Univariable Analysis		Multivariable Analysis	
Predictor	RR (95% CI)	*p*-Value	RR (95% CI)	*p*-Value
**PWH**				
Global T-score ^a^	0.916 (0.848, 0.990)	**0.027**	0.907 (0.840, 0.981)	**0.012**
Age (decades) ^a^	1.14 (1.07, 1.21)	**<0.001**	1.13 (1.07, 1.21)	**<0.001**
BMI	1.004 (0.994, 1.015)	0.41	1.008 (0.997, 1.018)	0.15
Sex	0.93 (0.77, 1.123)	0.45	-	-
Race/Ethnicity		**0.003**		**0.005**
Hispanic (vs. Black)	0.734 (0.592, 0.91)	**0.005**	0.722 (0.582, 0.896)	**0.003**
White (vs. Black)	1.04 (0.888, 1.219)	0.62	0.988 (0.843, 1.159)	0.89
Other (vs. Black)	1.187 (0.836, 1.687)	0.34	1.174 (0.829, 1.663)	0.37
Nadir CD4 ^b^	1.004 (0.994, 1.013)	0.45	-	-
plasma HIV RNA ^c^	0.989 (0.946, 1.035)	0.65	-	-
Myocardial infarction	1.483 (1.162, 1.82)	**0.002**	1.391 (1.092, 1.772)	**0.007**
Hyperlipidemia	1.19 (1.055, 1.342)	**0.005**	-	-
ART regiment type	-	0.45	-	-
**PWoH**				
Global T-score ^a^	0.994 (0.977, 1.012)	0.50	0.995 (0.978, 1.013)	0.58
Age (decades) ^a^	1.26 (1.13, 1.39)	**<0.001**	1.305 (1.16, 1.438)	**<0.001**
BMI	1.01 (0.994, 1.026)	0.24	1.019 (1.003, 1.036)	**0.02**
Sex	1.022 (0.79, 1.324)	0.87	-	-
Race/Ethnicity		0.52	-	-
Hispanic (vs. Black)	0.923 (0.629, 1.354)	0.68	-	-
White (vs. Black)	0.813 (0.588, 1.126)	0.21	-	-
Other (vs. Black)	0.68 (0.297, 1.56)	0.36	-	-
Nadir CD4 ^b^	1.006 (0.978, 1.035)	0.66	-	-
Myocardial infarction	1.614 (0.86, 3.028)	**0.14**	-	-
Hyperlipidemia	1.713 (1.366, 2.148)	**<0.001**	1.59 (1.313, 2.243)	**<0.001**

^a^ The unit is 10; ^b^ square root and ^c^ log10 transformation; BMI = body mass index; RR = rate ratio.

**Table 3:** Association between sRCS and MAP using linear mixed-effects models with both random slope and intercept, stratified by HIV status.

Outcome	Predictor	Coefficient (95%CI)	*p*-Value
**PWoH**			
Univariable M1: MAP	sRCS	−0.70 (−4.19, 2.80)	0.70
Multivariable M2: MAP ^c^	sRCS	−0.70 (−4.19, 2.80)	0.70
**PWH**			
Univariable M3: MAP	sRCS	−1.25 (−2.35, −0.15)	**0.027**
Multivariable M4: MAP	sRCS	−1.10 (−2.20, 0.00)	**0.0498**
	BMI	0.23 (0.11, 0.34)	**<0.001**
	Sex (ref. male)	−1.78 (−3.83, 0.27)	0.090
	Race/Ethnicity		
	Hispanic (ref. Black)	−3.08 (−5.31, −0.85)	**0.007**
	White (ref. Black)	−3.41 (−5.11, −1.72)	**<0.001**
	Other (ref. Black)	−2.55 (−6.32, 1.22)	0.18
	plasma HIV RNA ^d^	0.592 (0.18, 1.01)	**0.005**

^c^ all potential covariates were removed from the multivariable model in HIV—due to *p* > 0.15, ^d^ log10 transformation.

**Table 4:** Assessment of association between global T-score and anticholinergic medications in a stratified analysis by HIV status using mixed-effects models, adjusting for covariates.

		PWH		PWoH	
Outcome	Predictor	ES (95% CI)	*p*-Value	ES (95% CI)	*p*-Value
**Global T-score**	anticholinergic count ^a^	−0.27 (−0.38, −0.15)	**<0.001**	0.07 (−0.26, 0.40)	0.68
	Race/Ethnicity	-	**<0.001**	-	**<0.001**
	Hispanic (vs. Black)	−1.52 (−2.03, −1.00)	**<0.001**	−2.37 (−3.50, −1.25)	**<0.001**
	White (vs. Black)	−1.15 (−1.54, −0.76)	**<0.001**	−1.77 (−2.73, −0.82)	**<0.001**
	Other (vs. Black)	−0.88 (−1.62, −0.14)	**0.02**	−2.75 (−4.16, −1.33)	**<0.001**
	Nadir CD4 ^a^	0.03 (0.01, 0.05)	**0.005**	-	-
	Plasma HIV RNA ^b^	−0.04 (−0.10, 0.01)	0.14	-	-
	BMI	-	-	0.04 (0.01, 0.07)	**0.004**
	Time		0.087	-	-

^a^ square root and ^b^ log10 transformation; ES = standardized coefficient; BMI = body mass index.

**Table 5:** The outcomes, main predictors, and potential covariates.

Type	Variable
Outcome	Number of antihypertensive medications, MAP
Main predictor	Global T-scores or sRCS
Potential covariates	Age, sex, ethnicity/race, BMI, nadir CD4 count, and HIV RNA levels

## Data Availability

Dataset available on request from the authors.

## Abbreviations

The following abbreviations are used in this manuscript:

PWH	People With HIV
HTN	Hypertension
PWoH	People without HIV
sRCS	Summary Regression-based Change Score
MAP	Mean Arterial Pressure
ART	Antiretroviral Therapy
CVD	Cardiovascular Disease
SBP	Systolic Blood Pressure
DBP	Diastolic Blood Pressure
MetS	Metabolic Syndrome
NCI	Neurocognitive Impairment
AD	Alzheimer’s Disease
CHARTER	CNS HIV Antiretroviral Therapy Effects Research
NC	Neurocognitive
HNRC	HIV Neurobehavioral Research Center
CLIA	Clinical Laboratory Improvement Amendments
Poisson GLMM	Poisson Generalized Linear Mixed Effect Models
BMI	Body Mass Index
CI	Cognitive Impairment
MCI	Mild Cognitive Impairment

## Appendix A

Category A includes asymptomatic HIV infection, persistent generalized lymphadenopathy, or acute HIV infection. Category B consists of symptomatic conditions that indicate immune dysfunction but do not meet AIDS-defining criteria. Category C includes AIDS-defining illnesses such as opportunistic infections (e.g., Pneumocystis jirovecii pneumonia, toxoplasmosis of the brain), HIV-related encephalopathy, and certain malignancies (e.g., Kaposi’s sarcoma, invasive cervical cancer). CD4+ T-cell categories are as follows: Category 1: ≥500 cells/µL, Category 2: 200–499 cells/µL, and Category 3: <200 cells/µL (severe immunosuppression, associated with AIDS).

**Table A1:** Distribution of CDC HIV Clinical Staging in PWH in the study population.

CDC Stage	Count	Proportion
A1	64	5.42%
A2	161	13.6%
A3	104	8.8%
B1	16	1.35%
B2	111	9.4%
B3	176	15%
C1	11	0.9%
C2	57	4.82%
C3	480	40.64%
